# New gait performance indices and cognitive functions: a pilot study on correlation in people with Parkinson’s disease

**DOI:** 10.3389/fnhum.2025.1636813

**Published:** 2025-11-13

**Authors:** Elena Sofia Cocco, Carrie-Louise Thouant, Luca Pietrosanti, Francesco Infarinato, Carlotta Maria Manzia, Paola Romano, Raimondo Stefano Maria Torcisi, Marco Franceschini, Cristiano Maria Verrelli, Sanaz Pournajaf

**Affiliations:** 1 Neuromotor Rehabilitation and Rehabilitation Robotics, IRCCS San Raffaele Roma, Rome, Italy; 2 Electronic Engineering Department, University of Rome Tor Vergata, Rome, Italy; 3 Rehabilitation Bioengineering Laboratory, IRCCS San Raffaele Roma, Rome, Italy; 4 Department of Mental and Physical Health and Preventive Medicine, University of Campania ‘Luigi Vanvitelli’, Naples, Italy

**Keywords:** Parkinson’s disease, gait analysis, cognitive functions, self-similarity, lower limb rehabilitation, golden ratio, Fibonacci sequence, walking gait

## Abstract

Parkinson’s disease (PD) is the second most common neurodegenerative disease in the world and involves impairment of both motor and cognitive functions, significantly affecting the quality of walking and consequently the quality of life of people affected by this disease. This study analyzed the relationship between gait alterations and cognitive deterioration, using validated clinical tests and an innovative indicator, the *φ*-bonacci gait number, which quantifies gait harmonicity, symmetry, and consistency. Kinematic data collected during the 6-Minute Walk test on 19 people with PD (pwPD) and 15 healthy adults were analyzed. The results highlighted a significant negative correlation between gait harmonicity and cognitive performance (*φ*-bonacci gait number—Time Up and Go Dual Task; *r* = 0.797, *p* < 0.05). Finally, mediation analysis showed that global cognitive function (MoCA) indirectly influences gait harmonicity through TUG-DT. The results suggest that gait in pwPD is strongly modulated by executive-attentional functions, supporting its cognitive modulation rather than a purely automatic nature. This study allowed to explore the complex relationship between cognitive functions and the motor system, deepening how these interactions influence and refine motor behavior. Therefore, rehabilitation programs combining motor exercises and cognitive training could be more effective in improving gait quality, reducing the risk of falls, and improving person’s quality of life.

## 1 Introduction

Parkinson’s disease (PD) is the second most common neurodegenerative disorder worldwide (Feigin et al., 2019). It is characterized by significant motor deficits, including postural instability, gait variability and progressive cognitive decline, particularly evident in advanced stages. These deficits make people particularly vulnerable during dual tasks requiring simultaneous motor and cognitive skills (Gaßner et al., 2017; Woollacott and Shumway-Cook, 2002). Motor and cognitive impairments severely affect the quality of life, increasing the risk of falls, functional dependency, and disability in individuals, thus highlighting the need for integrated rehabilitation strategies (O’Shea et al., 2002; Roheger et al., 2018). According to the International Classification of functioning, disability and health (ICF) framework (Stucki et al., 2002), PD affects multiple domains, including body function, activities, and participation, which is crucial for designing comprehensive rehabilitation interventions (Stucki et al., 2002; Capecci et al., 2019). Baseline motor and cognitive impairment are likely predictors of more rapid motor decline and disability. Furthermore, further difficulty is linked to the variability of symptoms caused by the adverse effects of drugs, including levodopa. Motor assessment of Parkinson’s disease can be performed through clinical assessments of balance and posture, arm and hand function, and gait (Goffredo et al., 2024; Opara et al., 2017). Clinical assessment tools are fundamental in evaluating the motor and cognitive abilities of individuals, providing essential insights to guide effective rehabilitation programs. The Hoehn and Yahr (H&Y), the Unified Parkinson’s Disease Rating Scale (UPDRS), the Timed Up and Go Test (TUG), the six-minute walking test (6MWT) are among the most used. However, it is important to distinguish between qualitative gait assessment, which is based on clinical observation, and quantitative gait assessment, which relies on instrumented tools that allow for objective measurement of various spatiotemporal parameters (Bailo et al., 2024; Tramontano et al., 2024). Currently, wearable technology has entered advances in the healthcare sector, demonstrating its effectiveness, particularly in gait analysis, access previously inaccessible parameters (Tsakanikas et al., 2023). Instrumentation for movement analysis, in particular inertial measurement units (IMUs), provide sensitive and objective measures, essential for monitoring motor behaviors (Espay et al., 2016; Cocco et al., 2024). The marker-based motion capture system (Ghattas and Jarvis, 2024) is the gold standard but is more complex and expensive than IMUs which offer a cost-effective, easy-to-use, environmentally friendly and non-invasive alternative (Cappozzo et al., 2005; Romano et al., 2021). Human walking is now understood to generates rhythmic motor patterns with hidden temporal harmonic structures reflected in the presence of the golden ratio as a ratio between specific gait subphase durations (Picerno et al., 2021). Harmonic proportions can be influenced by neurological disorders, thus drastically reducing the smoothness, well-synchronized flow of movements, and alterations in gait self-similarities (El Arayshi et al., 2022). Specifically, in people with PD (pwPD), which is known to be characterized by resting tremor, rigidity, akinesia, or bradykinesia and postural instability, the fluid and rhythmically consistent flow of movement is reduced, and gait self-similarity is altered (Iosa et al., 2016). Recently, the *φ*-bonacci gait number and the concept of Harmonic Gait Variability (HGV), were introduced, enabling the quantitative measurement of the gait deviation from a harmonic avatar model (Verrelli et al., 2021). Such tools help clinicians evaluate the recursion, asymmetry, coherence, and harmonicity of the gait cycle along with the harmonicity noise over the gaits while offering valuable data to guide rehabilitation interventions. By incorporating the concept of ‘activities’ from the ICF (Stucki et al., 2002), this study aims to assess how gait impairments impact mobility and daily function. Since investigating interference effects in single-task and dual-task conditions in pwPD can provide valuable information on the severity of motor impairments, this approach can clarify how PD affects both motor and cognitive domains. Nevertheless, studies with dual tasks such as the TUG Dual-Task (TUG-DT) are fundamental for understanding the effects of motor-cognitive interference because they simulate real-life scenarios in which people must simultaneously manage motor and cognitive tasks, such as walking while talking (Klotzbier et al., 2025). The starting hypotheses of this study is that a lower smoothness of walking is associated with worse scores in cognitive tests, which could suggest that cognitive impairment is correlated to automatic control of walking, making pwPD more dependent on attentional resources for walking. Furthermore, if the TUG-DT significantly penalizes performance compared to the TUG in people with low harmonicity of gait, it is hypothesized that these individuals have difficulty in compensating for the cognitive load during movement, increasing the risk of instability. Furthermore, in the case of people with reduced gait harmonicity, a worse performance in the dual-task may indicate greater competition between motor and cognitive resources, suggesting an altered integration between the two domains.

The focus of this observational pilot study is on the analysis of gait in pwPD, evaluating the specific characteristics mentioned above—recently explored—in relation to cognitive functions. This is made possible by the biomechanical assessment and exploration of the *φ*-bonacci gait number for the evaluation of harmonic gait. In this regard, it is essential to analyze how any cognitive deterioration due to PD influences motor ability during walking, highlighting the implications of cognitive-motor interactions in the clinical setting. The objective is to investigate whether gait imbalances are related to motor coordination and cognitive disorders, using validated clinical tests such as the TUG and the TUG-DT, the 6MWT, the MDS-UPDRS and the Montreal Cognitive Assessment (MoCA). This exploration considers how cognitive impairments and motor coordination deficits interact to influence motor performance. In line with the ICF domains of “body functions” and “activities,” it has been examined how cognitive decline influences motor behavior and how rehabilitation might address these dual deficits.

## 2 Materials and methods

### 2.1 Study design

This cross-sectional observational study evaluated the motor performance of person with Parkinson’s disease compared to healthy adults during a walking test to investigate how cognitive and motor coordination deficits interact to affect gait function. This study adhered to the Declaration of Helsinki and was approved by the local ethics committee (no PR. 21/30 of December 2021).

### 2.2 Participants

Twenty-two persons with PD were recruited from the day-hospital of the IRCCS San Raffaele (Rome, Italy) as experimental group (EG) and fifteen healthy adults were enrolled as a control group (CG). All eligible participants signed informed consent and met the inclusion criteria set out in the study protocol. Inclusion criteria for the EG included: age between 30 and 80 years; diagnosis of Parkinson’s disease according to the Movement Disorder Society (MDS); Hoehn & Yahr (H&Y) scale score between 2 and 3 in the “on” phase; Montreal Cognitive Assessment (MoCA) screening test score ≥22 (Fiorenzato et al., 2024); Movement Disorder Society Unified Parkinson’s Disease Rating Scale (MDS-UPDRS) Part IV score ≤2 in both items (duration and disability) related to dyskinesias; stabilized treatment; ability to understand and sign the informed consent for the study; ability to comply with the study procedures. Exclusion criteria included: neurological pathologies superimposed on Parkinson’s disease, psychiatric complications, or personality disorders; musculoskeletal disorders that compromise movement; severe language deficits that may lead to the inability to understand and comply with the study procedures; absence of signed informed consent to the study. Inclusion criteria for the CG included age between 35 and 85 years old, absence of pathologies affecting the lower limb function and cognitive and/or severe visual deficit. Although twenty-two subjects were initially recruited for the EG, valid and analyzable data were ultimately obtained for only nineteen participants. Two participants were unable to complete the 6-Minute Walk Test for clinical reasons (fatigue and discomfort), and one participant was excluded due to a technical issue during the temporal signal acquisition.

#### 2.2.1 Demographic and clinical assessments

The following data were recorded for each participant: age, sex, weight, height, date of diagnosis of disease, drug therapy, time of taking the last drug before carrying out the walking test. Overall disease-related disability was assessed using different clinical rating scales. Specifically, the MDS-UPDRS total and subtotal (scores part I, II, III, IV), H&Y, MoCA, Mini Balance Evaluation Systems Test (Mini-BEST test), 6MWT, TUG, TUG-DT, 10-meter Walk Test (10MWT), New Freezing of Gait Questionnaire (NFOG-Q), Activities-Specific Balance Confidence Scale (ABC) were performed. All outcome measures were collected in the “ON medication” phase i.e., 1 h after oral consumption of the usual Levodopa dose and always in the morning to minimize variability (Fahn, 2000), by highly specialized clinical staff of the IRCCS San Raffaele in Rome.

### 2.3 Experimental setup

**Figure 1:**
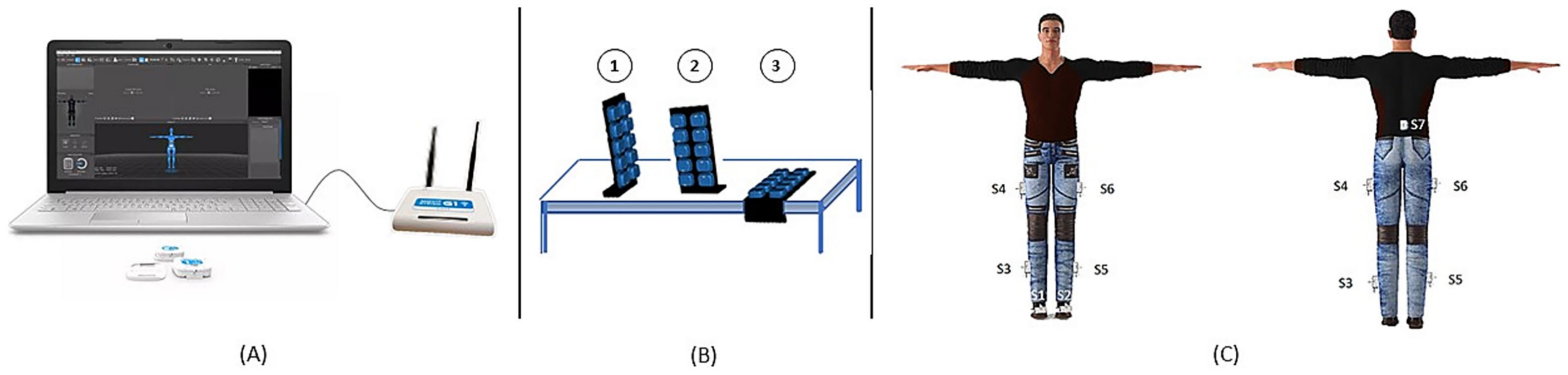
Experimental setup and instrumental assessment steps. **(A)** IMUs MOVIT network (Captiks s.r.l.); **(B)** first calibration phase on three positions; **(C)** second calibration phase—“T” pose.

The study was conducted at IRCCS San Raffaele (Rome, Italy) equipped with the IMUs MOVIT network (Captiks s.r.l., Rome, Italy) (Saggio et al., 2021). Each participant completed the 6MWT along a 15-meter corridor (Ngueleu et al., 2024; Cheng et al., 2020) with the inertial sensor equipment (Figure 1A). Each MOVIT (dimensions: width 48 mm, height 39 mm, depth 18 mm; weight: 40 g) hosts several sensors, a triaxial accelerometer and a triaxial gyroscope and, in addition to the raw data from the accelerometer and gyroscope, provides its orientation in space via a 6 degrees of freedom (6 DoF) quaternion. Thanks to a patented two-step calibration, the orientation of each sensor can be mapped with that of the body joints, allowing the reconstruction of kinematics and anatomical angles. The first calibration step (Figure 1B) involves acquiring three positions through two 90° rotation movements using a calibration base, in order to define a unique reference system. The second step (Figure 1C) consists of acquiring the “T” pose (standing with arms extended parallel to the ground) to align the MOVIT system with the body’s coordinate system. Seven inertial sensors were placed on the trunk (at lumbar vertebra L5), the lateral mid-third of the femur, the lateral mid-third of the fibula, the dorsal surface of the foot on both the right and the left lower limb (Figure 1C). Sensors were secured using Velcro straps over the participants’ clothing, which was selected to ensure strap stability during the walking test. The MOVIT system was validated using a video-based reference system (Saggio et al., 2021) (Vicon, Oxford Metrics), demonstrating excellent accuracy and repeatability with RMSE errors in joint angles below 3.5°.

### 2.4 Signal processing

The data acquisition and processing system (Captiks s.r.l., Rome, Italy) includes two core software components: the Motion Studio, used for session recording and system configuration, and the Motion Analyzer, for post-session data analysis. Data processing involves a temporal analysis on a portion of gaits at the centre of the straight path. Specifically, the system identifies time instants corresponding to key gait events corresponding to right heel strike, left toe-off, left heel strike, and right toe-off, across two successive gait cycles. These events are obtained using a custom extraction algorithm developed for this purpose. The raw data (.csv file) for each sensor is downloaded via Motion Studio, then loaded into the Motion Analyzer to extract heel strike and toe-off timing for the left and right foot, as well as the joint angles. These data are then analyzed through a dedicated MATLAB to determine gait cycle (GC) boundaries and subphases, which are essential for computing the *φ*-bonacci gait number.

**Figure 2:**
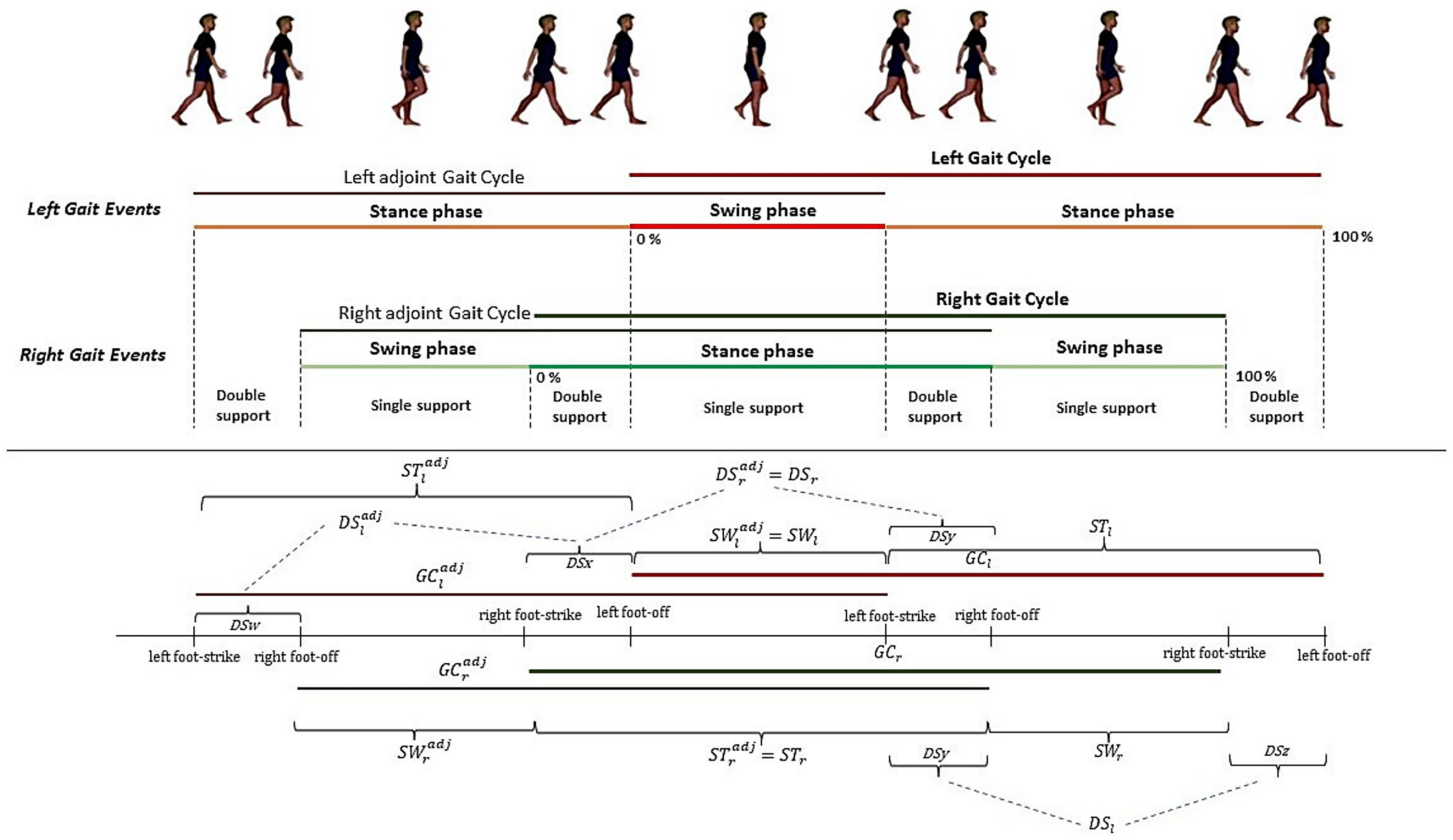
Composite gait cycle: right and left gait events and adjoint right and left gait cycles.

To understand this metric, it is important to define the concept of gait cycle and gait sub-phases. A single gait cycle is defined as the interval between two consecutive heel strike of one foot (for the sake of simplicity, we consider the right foot as reference, however the following discussion can be easily adjusted for the left foot). We can divide the gait cycle according to heel strike and toe off time instants as follows: the time between a heel strike and toe off is called Stance Phase (ST), while the time between the toe off and the next heel strike is named Swing Phase (SW). Moreover, if we consider both feet, we can define the Double Support phase (DS) as the time occurring between the heel strike of one foot and the toe off of the other foot. A graphical explanation is reported in Figure 2.

As previously demonstrated in (Verrelli et al., 2021) the sequence DS, SW, ST, GC form a finite length Fibonacci sequence. This property can be used to defines a set of quantities, each evaluating a specific aspect of gait:

$$A_{1}=\sqrt{{\left(\frac{{\mathrm{SW}}_{l}}{{\mathrm{DS}}_{r}}-\phi \right)}_{n}^{2}+{\left(\frac{{\mathrm{SW}}_{r}}{{\mathrm{DS}}_{l}}-\phi \right)}_{n}^{2}+\mu^{\mathrm{adj}}{\left(\frac{{\mathrm{SW}}_{r}^{\mathrm{adj}}}{{\mathrm{DS}}_{l}^{\mathrm{adj}}}-\phi \right)}_{n}^{2}}$$ (1)

$$A_{2}=\lambda \sqrt{{\left(\frac{{\mathrm{SW}}_{r}}{{\mathrm{SW}}_{l}}-1\right)}_{n}^{2}+\lambda^{\mathrm{adj}}{\left(\frac{{\mathrm{SW}}_{r}^{\mathrm{adj}}}{{\mathrm{SW}}_{r}}-1\right)}_{n}^{2}}$$ (2)

$$A_{3}=\delta \sqrt{{\left(\frac{{\mathrm{DS}}_{x}}{{\mathrm{DS}}_{y}}-1\right)}_{n}^{2}}$$ (3)

where the subscripts *r*, *l* are referred, respectively, to right and left feet, and *adj* means the adjoint gait cycle, i.e., the next (or previous) gait cycle of the opposite foot, μ,λ,δ are weights that can be adjusted according to the specific case (here we use a weight of 1 for all), and the subscript *n* is related to the following normalization formula (Equation 4):

$${\left(\frac{\xi_{n}}{\xi_{\mathrm{ed}}}-\xi_{v}\right)}_{n}^{2}=(\frac{\xi_{d}}{\xi_{n}}){\left(\frac{\xi_{n}}{\xi_{\mathrm{ed}}}-\xi_{v}\right)}^{2}$$ (4)

More in detail, *A*1 is associated with the self-similarity of gait, *A*2 represent the SW symmetry between left and right feet, and *A*3 defines DS “consistency.”

From Equations 1–3 in an ideal gait characterized by high self-similarity, symmetry, and consistency, *A*1, *A*2 and *A*3 are zero.

Combining Equations 1–3 we can define the *φ*-bonacci gait number as the sum of A1, A2, A3, namely (Equation 5):

$$\begin{array}{l} \phi_{b}=\sqrt{{\left(\frac{{\mathrm{SW}}_{l}}{{\mathrm{DS}}_{r}}-\phi \right)}_{n}^{2}+{\left(\frac{{\mathrm{SW}}_{r}}{{\mathrm{DS}}_{l}}-\phi \right)}_{n}^{2}+\mu^{\mathrm{adj}}{\left(\frac{{\mathrm{SW}}_{r}^{\mathrm{adj}}}{{\mathrm{DS}}_{l}^{\mathrm{adj}}}-\phi \right)}_{n}^{2}}\\ +\lambda \sqrt{{\left(\frac{{\mathrm{SW}}_{r}}{{\mathrm{SW}}_{l}}-1\right)}_{n}^{2}+\lambda^{\mathrm{adj}}{\left(\frac{{\mathrm{SW}}_{r}^{\mathrm{adj}}}{{\mathrm{SW}}_{r}}-1\right)}_{n}^{2}}+\delta \sqrt{{\left(\frac{{\mathrm{DS}}_{x}}{{\mathrm{DS}}_{y}}-1\right)}_{n}^{2}} \end{array}$$ (5)

which encapsulates all aspects of gait in a single index.

In summary, the *φ*-bonacci number for gait integrates three fundamental features of locomotion. First, self-similarity (*A*1) characterizes gait harmonicity as a measure of how much the larger scale structure resembles the subunit structure, within an internal evolutionary process including the generation of a self-referential loop. Second, symmetry (*A*2) quantifies the balance between left and right limb swing phases, capturing bilateral coordination. Third, DS “consistency” (*A*3) assesses the stability of the double-support phases across sub-cycles, indexing the temporal invariance of transitions between limbs. In an ideal, highly harmonious gait, these deviations are minimized and the *φ*-bonacci number approaches zero, while higher values are related to irregularity, asymmetry, or instability in gait timing. Unlike conventional spatiotemporal descriptors, which provide isolated measures (e.g., step time, stance duration, cadence), the *φ*-bonacci index encapsulates the multidimensional harmonic organization of gait in a single composite index.

### 2.5 Statistical analysis

Statistical analysis of the data was performed using Matlab R2023b (The MathWorks, Natick, MA, USA) as the computing and programming platform. For the described demographic and clinical data of the sample, frequencies with relative percentage, mean value with standard deviation, and median value were calculated for the categorical, continuous, and ordinal variables, respectively. Inferential analysis was conducted using the Kolmogorov–Smirnov test to evaluate the normality of the data distributions. The dual-task cost (DTC) was computed as (TUG-DT − TUG)/TUG × 100, providing a normalized percentage change between single- and dual-task conditions. Univariate and multivariate analyses were performed to explore the associations between the *φ*-bonacci gait number and the collected clinical variables. A multiple linear regression model was first fitted including all clinical predictors (TUG, TUG-DT, UPDRS II, UPDRS III, MoCA). Stepwise selection was then applied using the Bayesian Information Criterion (BIC) as a stopping rule. At each step, variables were either included or excluded based on their contribution to the model fit. The final model retained TUG-DT as the only significant predictor. For all models, regression coefficients (*β*), standard errors (SE), 95% confidence intervals (CI), *t* statistics, degrees of freedom, and *p*-values were reported. Both unadjusted and HC3 heteroskedasticity-robust standard errors were calculated. The ordinary least squares (OLS) assumptions were tested: residual normality (Shapiro–Wilk, QQ plots), homoskedasticity (Breusch-Pagan), linearity and multicollinearity (VIF). Influential observations were assessed using Cook’s distance (threshold 4/n) and Studentized residuals. HC3 heteroskedasticity-robust standard errors were observed, and a sensitivity analysis was conducted excluding influential cases. Correlations were calculated through the Spearman coefficient between the *φ*-bonacci gait number and the clinical scales, and an alpha value of 0.05 was set for all statistical analyses. To investigate a possible mediation effect of the TUG-DT in the relationship between cognitive abilities assessed through the MoCA and gait harmonicity expressed through the *φ*-bonacci gait number, a non-parametric mediation analysis was performed with a bootstrap procedure (He et al., 2024) through an algorithm developed in Matlab setting a fixed seed for reproducibility [rng(42)]. Bootstrapping was used to test the mediation effect, with a duplicate sample size of 2000 (He et al., 2024). If the 95% confidence interval of the indirect effect did not include zero, the mediation effect was shown to be significant. The indirect effect was estimated as the product of the coefficients of the two linear regressions: regression of the TUG Dual Task mediator on the MoCA variable and regression of the *φ*-bonacci gait number variable on the mediator, controlling for the MoCA variable. All spatiotemporal parameters were compared between groups using the non-parametric Mann–Whitney U test (*α* = 0.05). To account for multiple comparisons across parameters, Bonferroni correction was applied.

## 3 Results

The study included 19 participants diagnosed with Parkinson’s disease (EG) and 15 healthy controls (CG). In the EG, the mean age was 72.27 years (±7.49), and 63.2% were male. The mean disease duration was 7.0 years (±2.62). In the CG, the mean age was 68.53 years (±3.56), and 60.0% were male. Table 1 describes all clinical and demographic characteristics of the sample. Disease severity, in the EG, assessed using the Hoehn and Yahr scale, had a median of 2.5, ranging from 2 to 2.5. Scores on the UPDRS revealed a moderate overall level of impairment, with median scores of 11 for section I (mental, behavioral, and emotional aspects), 9.5 for section II (activities of daily living), 11 for section III (motor examination), and 4 for section IV (treatment-related complications), with a total median UPDRS score of 38 (range 15–79). Regarding quality of life, measured by the PDQ-8 questionnaire, the EG participants reported a moderate impact, with a median score of 5.5. Cognitive functions were relatively well preserved, as reflected by the median of MoCA. Balance and mobility abilities were assessed using the Mini-BESTest and various gait tests. The EG had a median score on the Mini-BESTest was 24.5 (ranging from 17 to 29). The average time to complete the TUG test was 10.27 s (±3.39) in the EG and 6.8 s (±2.91) in the CG. Under dual-task conditions, the EG averaged 13.23 s (±4.93), compared to 11.9 s (±1.54) in the CG. The 6MWT distance was 394.67 m (±0.11) in the EG, significantly shorter than the CG (500.09 m ±18.33). The 10MWT was completed in 6.42 s (±0.99) in the EG and 6.1 s (±0.23) in the CG. Finally, freezing of gait episodes, as assessed with the NFOG-Q, had a median score of 5 (ranging from 0 to 13). Balance confidence, assessed using the ABC scale, showed a median of 82.19%, with values ranging from 19 to 95.6%, reflecting generally high confidence with notable variability among participants.

**Table 1:** Demographic and clinical characteristics of the enrolled study participants.

Participants characteristics (EG = 19; CG = 15)		
Demographic and clinical characteristics	EG	CG
Age (years)	72.27 ± 7.49	68.53 ± 3.56
Gender Male, *n* (%)	12 (63.2%)	9 (60.0%)
Disease onset (years)	7.0 ± 2.62	–
H&Y	2.5 (2–2.5)	–
UPDRS I	11 (3–30)	–
UPDRS II	9.5 (4–23)	–
UPDRS III	11 (5–21)	–
UPDRS IV	4 (0–13)	–
UPDRS TOT	38 (15–79)	–
PDQ-8	5.5 (0–16)	–
MoCA	28 (22–29)	–
Mini-BESTest	24.5 (17–29)	–
TUG (sec)	10.27 ± 3.39*	6.8 ± 2.91*
TUG Dual-Task (sec)	13.23 ± 4.93*	11.9 ± 1.54*
6MWT (m)	394.6 ± 70.11*	500.09 ± 18.33*
10mWT (sec)	6.42 ± 0.99	6.1 ± 0.23
NFOG-Q	5 (0–13)	–
ABC (%)	82.19 (19.0–95.6) %	–

Data are reported as mean ±standard deviation or frequency with percentage (%) or median (range min–max). *p < 0.05 (Mann–Whitney U test; statistically significant differences between groups).

EG, Experimental Group; CG, Control Group; H&Y, Hoehn & Yahr; UPDRS, Unified Parkinson’s Disease Rating Scale; PDQ-8, Parkinson’s Disease Questionnaire–8 items; MoCA, Montreal Cognitive Assessment; Mini-BESTest, Mini Balance Evaluation Systems Test; TUG, Time Up and Go Test; 6MWT, 6 Minute Walk Test; 10mWT, 10 meters Walk Test; NFOG-Q, New Freezing of Gait Questionnaire; ABC, Activities-specific Balance Confidence Scale.

The gait analysis results (Table 2) confirm findings reported in previous literature (Herssens et al., 2018). Comparing the space–time parameters obtained from the gait analysis of pwPD (EG), statistically significant differences emerge when compared with the same parameters in the normative case of healthy adult subjects who fall within the same age range of the recruited sample (CG). Specifically, stride length and speed (expressed, respectively, in m and m/s) seem to be two predictors of bradykinesia (Chien et al., 2006). Furthermore, the sample examined confirms how important it is to know exactly the individual’s state (i.e., “ON” or “OFF” phase) which varies with the temporal distance from the pharmacological therapy (Levodopa) and the results obtained confirm how in the “ON” phase the most effective predictor is the stride length (Chien et al., 2006).

**Table 2:** Spatiotemporal parameters results.

Spatio-temporal parameters								
Parameters	EG		CG		*p*-value		*p*-value (bonf)	
	Right	Left	Right	Left	Right	Left	Right	Left
Stride time (sec)	1.32 ± 0.47**	1.36 ± 0.55**	1.10 ± 0.02**	1.03 ± 0.02**	1.86 × 10^−4^	1.05 × 10^−6^	0.010	0.23 × 10^−3^
Stance phase time (sec)	0.84 ± 0.47**	0.85 ± 0.56**	0.69 ± 0.06**	0.69 ± 0.07**	3.95 × 10^−4^	1.50 × 10^−4^	0.023	0.009
Swing phase time (sec)	0.48 ± 0.07*	0.49 ± 0.04*	0.42 ± 0.01*	0.42 ± 0.01*	3.85 × 10^−3^	1.69 × 10^−3^	0.227	0.099
Single support time (sec)	0.44 ± 0.02*	0.46 ± 0.08*	0.42 ± 0.01*	0.41 ± 0.01*	0.046	0.002	0.100	0.122
Double support time (sec)	0.42 ± 0.51**	0.44 ± 0.52**	0.27 ± 0.07**	0.27 ± 0.07**	3.64 × 10^−5^	3.87 × 10^−5^	0.002	0.002
Step time (sec)	0.67 ± 0.46**	0.65 ± 0.30**	0.56 ± 0.07**	0.55 ± 0.07**	5.23 × 10^−5^	0.92 × 10^−3^	0.003	0.045
Stance phase (%)	61.49 ± 4.98	57.94 ± 3.04	62.02 ± 0.96	62.01 ± 1.23	0.463	0.499	–	–
Swing phase (%)	37.08 ± 3.49	36.56 ± 2.72	37.99 ± 0.96	38.02 ± 1.26	0.445	0.207	–	–
Single support (%)	35.60 ± 1.37*	36.00 ± 2.14	37.99 ± 1.27*	38.02 ± 1.06	0.011	0.109	0.682	–
Double support (%)	28.73 ± 2.38*	28.66 ± 2.79*	23.98 ± 1.52*	23.97 ± 1.53*	0.012	0.013	0.741	0.805
Step time (%)	50.86 ± 2.39	49.14 ± 3.42	50.01 ± 0.80	50.03 ± 0.86	0.378	0.167	–	–
Stride length (m)	0.76 ± 0.06**	0.77 ± 0.06*	1.24 ± 0.03**	1.04 ± 0.02*	0.68 × 10^−3^	0.014	0.040	0.854
Step length (m)	0.40 ± 0.03**	0.44 ± 0.03*	0.64 ± 0.02**	0.60 ± 0.02*	0.53 × 10^−3^	0.003	0.031	0.177
Speed (m/s)	0.62 ± 0.06**		1.14 ± 0.04**		0.68 × 10^−3^		0.020	
Cadence (step/min)	98.43 ± 14.22**		109.77 ± 5.15**		1.15 × 10^−3^		0.039	

Data are reported as mean ± standard deviation. *p < 0.05 uncorrected; **p < 0.05 Bonferroni-corrected (Mann–Whitney U test, comparisons performed separately for right and left side).

The calculated *φ*-bonacci gait number (Table 3) shows that its average trend in the neurological population differs significantly from the normative value examined on the healthy adult population. Compared to controls, *A*1, *A*2 and *A*3 were markedly altered in pwPD, whereas in the normative condition these parameters approach zero, reflecting optimal gait harmonicity.

**Table 3:** Walking indexes results.

Walking indexes				
Indexes	EG	CG	*p*-value	*p*-value (bonf)
*φ*-bonacci gait number	1.86 ± 0.82**	0.86 ± 0.26**	2.07 × 10^−6^	0.83 × 10^−6^
A1	0.58 ± 0.12**	0.03 ± 0.02**	0.88 × 10^−6^	0.35 × 10^−3^
A2	0.16 ± 0.11**	0.003 ± 0.003**	0.002	0.009
A3	1.04 ± 0.87**	0.21 ± 0.14**	0.79 × 10^−6^	0.31 × 10^−4^
GC	1.16 ± 0.09*	1.01 ± 0.01*	0.045	0.236

Data are reported as mean ± standard deviation. *p < 0.05 uncorrected; **p < 0.05 Bonferroni-corrected (Mann–Whitney U test).

**Figure 3:**
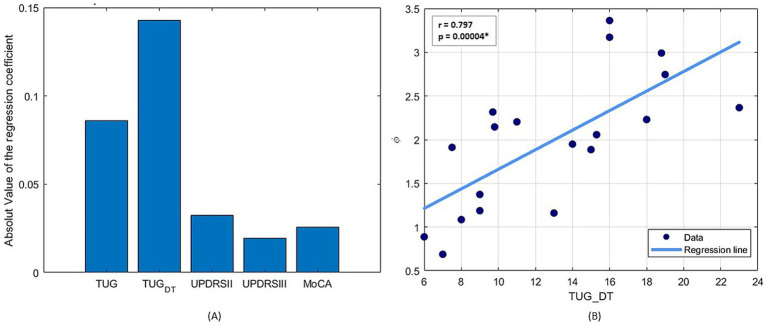
**(A)** Absolute value of the coefficients of the multiple linear regression to estimate the simultaneous relationship of clinical variables on *φ*-bonacci gait number. **(B)** Spearman linear correlation results between TUG Dual Task and *φ*-bonacci gait number.

Statistical analysis using multiple linear regression, followed by stepwise regression, was conducted to identify which clinical assessment between the TUG, the TUG-DT, UPDRS II and III and MoCA is most closely associated with *φ*-bonacci gait number. Among these, only the TUG-DT emerged as a statistically significant predictor, as indicated by the regression coefficients of the linear regression model in Figure 3A. All assumptions for multiple linear regression were checked. Tests confirmed residual normality (Shapiro–Wilk *p* = 0.84; Anderson–Darling *p* = 0.92) and homoscedasticity (Breusch–Pagan *p* = 0.34). No relevant multicollinearity was detected (all VIF < 3.5). One subject showed a Cook’s distance above the 4/n threshold, indicating a potential influential case. The overall OLS model (*R*^2^ = 0.42) identified TUG-DT as the only significant predictor of *φ* (*β* ≈ 0.13, *p* = 0.026 with robust HC3 SE), while other predictors were not significant. Concerning the sensitivity analysis, excluding the influential case, results were consistent (*R*^2^ = 0.42), and TUG-DT remained the only significant predictor (*β* ≈ 0.13, *p* = 0.015 OLS; *p* = 0.026 HC3-robust). All diagnostic tests remained satisfactory. The initial multiple regression model included TUG, TUG-DT, UPDRS II, UPDRS III, and MoCA. Stepwise selection (BIC criterion) progressively excluded UPDRS II, UPDRS III, MoCA, and TUG. The final model retained TUG-DT as the only significant predictor of *φ* (*β* = 0.127, SE = 0.050, *t* = 2.55, df = 13, *p* = 0.026). The adjusted *R*^2^ of the final model was 0.42. The overall *F*-statistic was not significant [*F*(5,13) = 2.03, *p* = 0.17] when all predictors were included, but the simplified model confirmed the unique predictive role of TUG-DT. Furthermore, from the Spearman linear correlation analysis between TUG-DT and *φ*-bonacci gait number (Figure 3B), a statistically significant medium-to-high positive correlation (Portney, 2020) (*r* = 0.797; *p* < 0.05) emerges. The fact that the TUG-DT was the only significant predictor of the *φ*-bonacci gait number suggests that it may represent an index sensitive not only to the motor component, but also to the contribution of executive and attentional functions necessary to preserve gait harmonicity in dual-task conditions. These findings support the main hypothesis of the study: the *φ*-bonacci gait number may be influenced by the contribution of cognitive function, demonstrating that motor behavior is critically influenced by cognitive processes such as attention, perception, and executive function.

**Figure 4:**
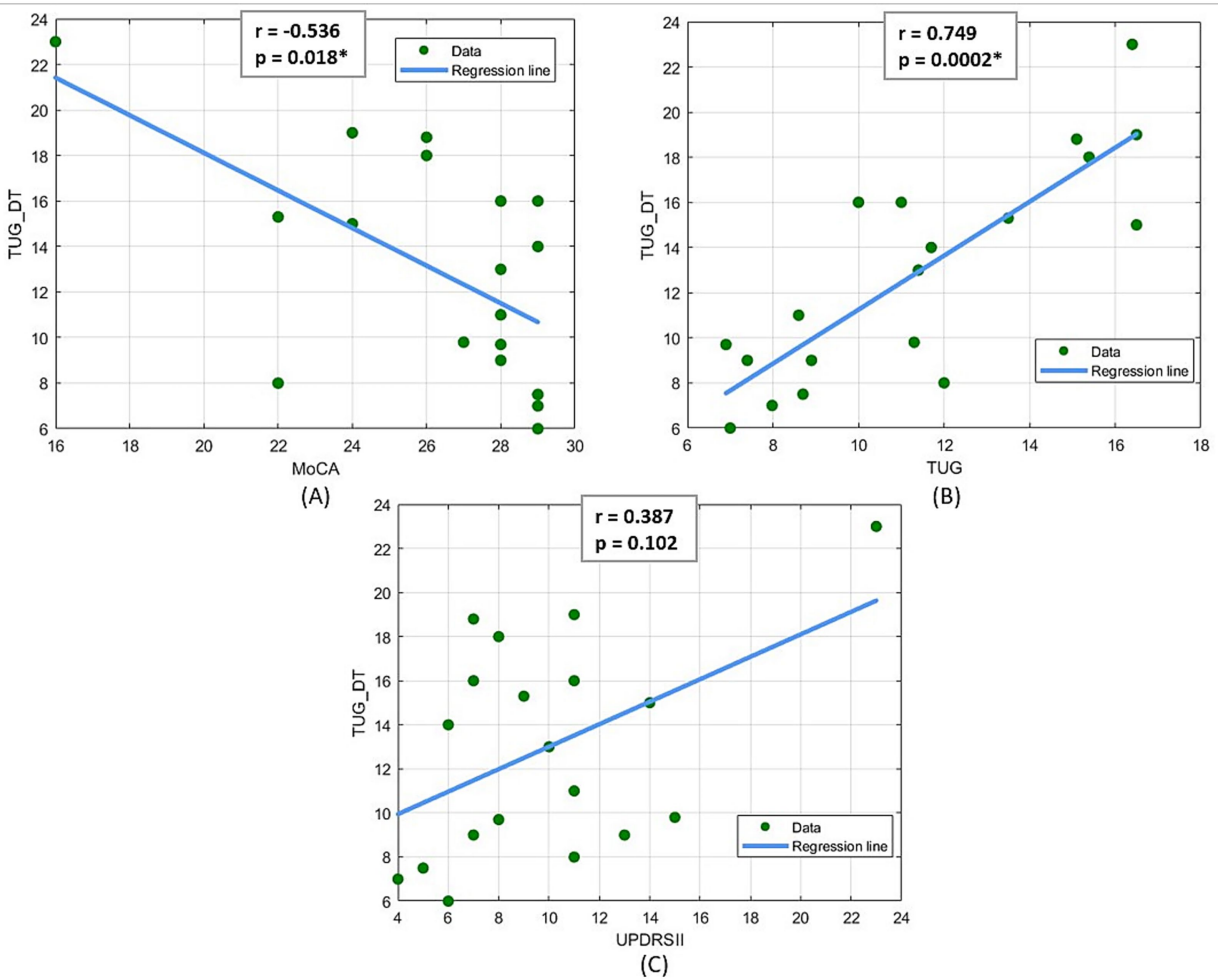
**(A)** Spearman linear correlation results between MoCA and TUG Dual Task, **(B)** Spearman linear correlation results between TUG and TUG Dual Task, **(C)** Spearman linear correlation results between UPDRS II and TUG Dual Task.

Given the significant aspect of the TUG-DT as an explanatory and objective clinical assessment, secondary correlation analyses were conducted using the Spearman coefficient to evaluate which of the other clinical scales used correlates more with the TUG-DT. The results of this analysis (Figure 4) demonstrate the existence of a statistically significant medium-to-high (Portney, 2020) negative correlation between TUG-DT and MoCA (*r* = −0.536; *p* < 0.05) and a high (Portney, 2020) positive correlation between TUG and TUG-DT (*r* = 0.749, *p* < 0.05). In contrast, no statistically significant correlations emerged between TUG-DT and the UPDRS parts II and III.

The dual-task cost (DTC) of the TUG was calculated to provide a normalized measure of motor–cognitive interference. Correlation analysis revealed a strong and statistically significant association between DTC and the *φ*-bonacci gait number (Spearman’s *r* = 0.782, *p* < 0.05), indicating that individuals exhibiting higher dual-task costs also demonstrated reduced gait harmonicity.

**Figure 5:**
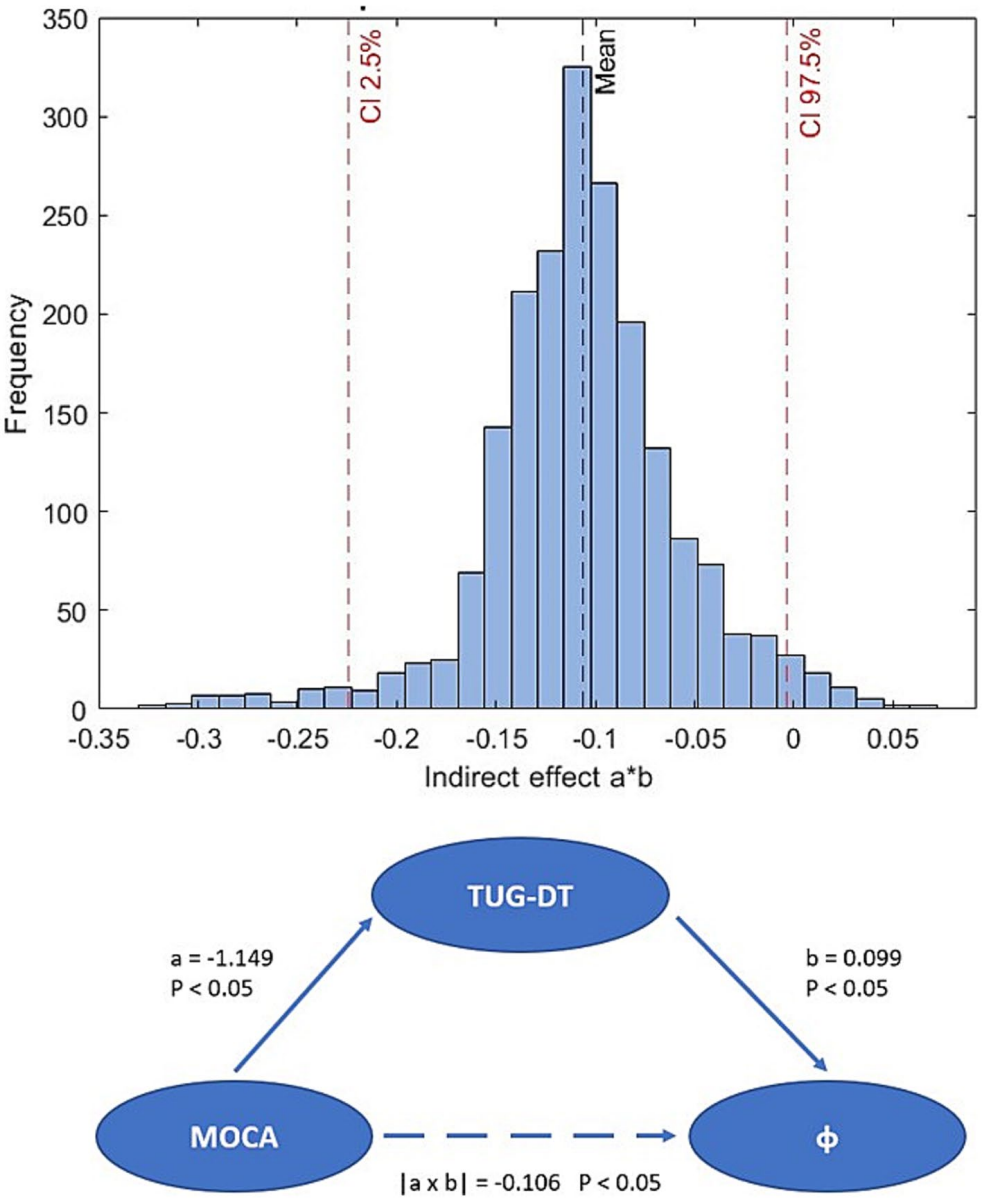
Bootstrap distribution of the indirect effect and bootstrap mediation scheme.

Starting from the fact that there is a significant correlation between TUG-DT and MoCA and wanting to investigate whether cognitive abilities can significantly influence walking motor performance, bootstrapping (He et al., 2024) was used to test the mediation effect (Figure 5), with a duplicate sample size of 2000. The 95% confidence interval of the indirect effect (a*b = −0.106) did not include zero (CI: −0.224; −0.003), therefore, the mediation effect proved to be significant. This technique was used to test the indirect effect of MoCA on *φ*-bonacci gait number. Mediation analysis showed that the MoCA score, indicative of global cognitive function, significantly and negatively predicted the time taken to complete the TUG-DT (*a* = −1.149; *p* < 0.05), suggesting that higher cognitive levels are associated with better motor performance in situations of increased attentional load. In turn, the duration of the TUG-DT was significantly and positively associated with the *φ*-bonacci gait number, controlling for the MoCA score (*b* = 0.099; *p* < 0.05), indicating that longer execution times correlate with lower gait harmonicity. The indirect effect of MoCA on *φ*-bonacci gait number, mediated by the TUG-DT, was equal to −0.106, and its significance was confirmed by bootstrapping with 2000 samples (95% CI not containing zero). These results may suggest that better cognitive function may contribute to improved gait harmonicity indirectly, by enhancing motor control in dual-task conditions.

**Figure 6:**
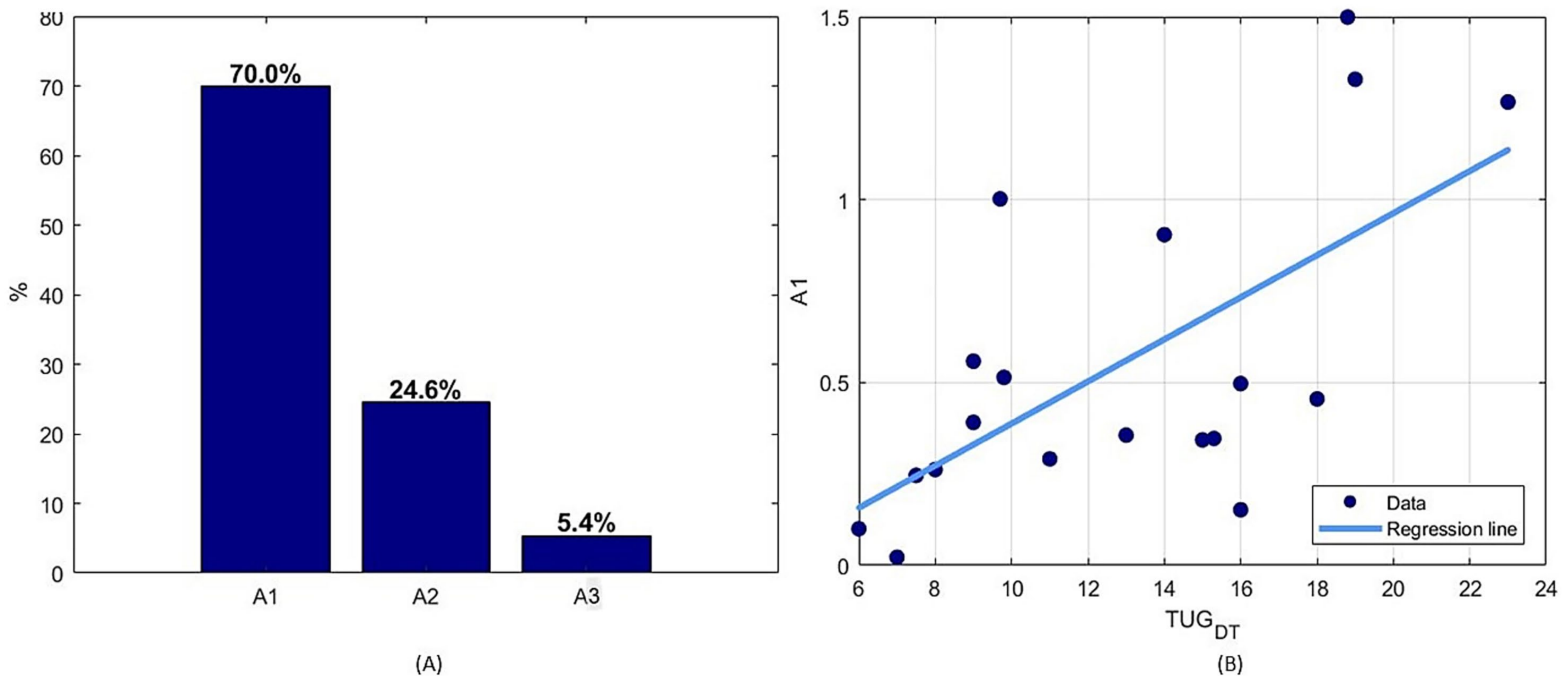
**(A)** Percentage normalized values of the coefficients of the multiple linear regression to estimate the simultaneous relationship of the set of parameters with respect to the *φ*-bonacci gait number, **(B)** Spearman linear correlation results between A1 parameter and TUG Dual Task.

Finally, given that the calculation of *φ* is influenced by multiple parameters (*A*1, *A*2, *A*3), the weight of each of these parameters with respect to the final value of *φ*-bonacci gait number was investigated in terms of percentage through a linear regression model. It emerged that the parameter *A*1, the index of self-similarity of gait, is the most influential parameter (Figure 6) and that it is statistically significantly correlated with the TUG-DT (*r* = 0.605, *p* < 0.05).

## 4 Discussion

### 4.1 Cognitive and motor interaction in Parkinson’s disease

This study aimed to analyze the impact that cognitive deficits, due to the presence of a neurological disease such as Parkinson’s disease, have on motor ability and how they influence it during walking. The objective was to investigate whether gait imbalances, quantitatively assessed through gait analysis, are related to motor coordination disorders and cognitive deficits, using validated clinical tests such as the TUG, TUG-DT, 6MWT, MDS-UPDRS and MoCA. The initial hypotheses of the study were confirmed by the fact that a lower gait fluency is associated with worse scores in cognitive tests. This result could suggest that cognitive deterioration compromises automatic control of walking, making pwPD more dependent on attentional resources for walking. It has also been confirmed that temporal harmonic features of walking are significantly disrupted by the PD, with marked reductions in movements fluidity, self-similarity, and gait smoothness (El Arayshi et al., 2022; Iosa et al., 2016). These results were achieved by analyzing the *φ*-bonacci gait number that allows the quantitative measurement of the deviation of the gait to a harmonic avatar model and that allows to monitor the recursion, asymmetry, coherence and harmonicity of the gait cycle, during walking. Kinematic data extracted during the 6MWT from 19 pwPD and 15 healthy adults age- and sex-matched meeting the study’s inclusion and exclusion criteria were examined. Among the clinical tests, the TUG-DT showed the stronger correlation with gait harmonicity, as measured by the *φ*-bonacci gait number, suggesting that these people may have difficulty compensating the cognitive load during movement, thereby increasing the risk of instability. Hence, TUG-DT should be considered an ecological test of motor–cognitive interaction. In people with worse harmonicity (Iosa et al., 2012), a lower performance in the dual task may also indicate a greater competition between motor and cognitive resources, suggesting a compromised integration between the two domains. Indeed, our results support the interpretation of the TUG-DT as a reliable probe of cognitive-motor interference, in line with meta-analytic evidence showing that walking performance worsens when a cognitive task is added. Specifically, examining all the parameters that influence *φ*-bonacci gait number calculation (*A*1, *A*2, *A*3) it was discovered that the one that has a dominant influence is the parameter *A*1, which is the descriptive factor of the self-similarity of gait. This confirms that the intrinsic harmonicity of gait is altered in pwPD and significantly correlated with motor severity, furthermore, it is demonstrated that the loss of harmonicity can be a quantitatively assessable reference parameter for gait in PD. Lastly, the observed negative correlation between TUG-DT and MoCA underscores the impact of cognitive decline on dual-task gait performance, reinforcing the role of cognitive function as a key determinant of functional mobility.

### 4.2 Fibonacci-based patterns as biomarkers of motor impairment

Further validation of our results comes from the comparison with previous work by Verrelli et al. (2021, 2025); healthy and pathological gaits were analyzed using generalized Fibonacci sequences and harmonization based on the golden ratio. The results showed that the pathological gait presents a reduced mechanization and a lower self-similarity compared to the healthy gait, with deviations from the ideal harmonic structure. Our data on pwPD fit this picture: the *φ*-bonacci values and the analyzed indices confirm that parkinsonian gait deviates from the harmonic organization based on the golden ratio, similarly to other pathological conditions. This comparison strengthens the idea that the loss of harmonicity is not only a phenomenon specific to Parkinson’s disease, but a general biomarker of motor dysfunction. Verrelli et al. (2025) proposed that cyclical human movements follow a dual process: temporal symmetrization. In healthy walking, this process allows for smooth, efficient, and low Shannon entropy movements. Our results demonstrate that this process is altered in pwPD: the *φ*-bonacci gait number and, in particular, the A1 parameter are altered and strongly correlated with the TUG-DT. This confirms that cognitive resources are essential for maintaining a harmonious gait, especially under dual-task conditions.

### 4.3 Spatio-temporal parameters and normative comparisons

All kinematic data and spatial–temporal parameters acquired during the 6MWT were collected and analyzed; these results were compared with normative data. Our results of the CG reflected the literature, specifically, a systematic review (Herssens et al., 2018) that collected 18 studies aimed at examining differences in spatial–temporal parameters and gait variability across the adult life span in healthy subjects. Data were extracted from 2,112 healthy adults. First, we observed that in pwPD, the average walking speed is characterized by a reduction of about 50% compared to healthy adults of the same age group. This could be dictated by the bradykinesia typical of the disease. Furthermore, the average step frequency expressed in steps/min observed in people with PD is lower than in healthy subjects of the same age group. This difference, greater than 10%, reflects a reduction in locomotor rhythm in affected subjects. Regarding step length, pwPD show significantly lower values than healthy adults. This corresponds to a reduction between 28 and 35%, indicative of a marked hypokinesia. The length of the double step (stride) in pwPD is also significantly shorter than in healthy subjects of the same age. This translates into an average reduction of approximately 37%, resulting in decreased movement efficiency between groups comparison and the results of the systematic review allowed us to demonstrate that pwPD have a significantly reduced walking speed, a lower cadence, a shortened step and stride length, a prolonged step time, and an increased double support, reflecting compensatory strategies to avoid falls. These differences highlight a marked reduction in gait efficiency and stability, characteristics of disease-related motor disorders. Furthermore, comparing these results with those obtained from a second study conducted by Beauchet et al. (2017) on a sample of healthy subjects of the same age, it was confirmed that the most significant parameter that differs most from the normative data is the stride length and then the speed expressed in m/s, and the results just described were also confirmed. However, it should be specified that these conclusions are valid when the person is in the “ON” phase, that is, approximately 1 h after taking the pharmacological therapy of Levodopa, and some parameters and results could be significantly variable if the participants is in the “ON” or “OFF” phase. Comparing the results obtained with those of the study by Chien et al. (2006) where data were acquired from people during both the “ON” and “OFF” phases who were following Levodopa as pharmacological therapy, the following emerged: between the two phases, a parameter that differs significantly emerges, which is the stride length, which could be used as the most effective predictor to determine the state of the pwPD when taking pharmacological therapy and when not taking it, therefore this parameter could be used to provide information on the degree of bradykinesia, unlike cadence which does not seem to vary between the two phases. Compared to the healthy subjects analyzed in the study by Chien et al. (2006), and comparing with the results obtained by us, the stride length and speed emerge as significant parameters of the presence of PD.

### 4.4 Assessing motor-cognitive interference through dual-task performance

More specifically, the correlation observed between the *φ*-bonacci gait number score, and the TUG-DT demonstrates the direct influence of the cognitive component on motor performance. It highlights the impact of a dual task on the harmonicity of the step during walking. In 2018, a meta-analysis analyzed 19 studies and the main results emerged through a random effects modeling: a strong negative effect of dual tasks on walking performance emerged, indicating that walking is significantly altered when a cognitive task is added (Beauchet et al., 2017; Yogev-Seligmann et al., 2008; Rochester et al., 2014; Maidan et al., 2016; Kehagia et al., 2013). These results confirm that dual tasks have an overall deleterious effect on walking in pwPD, highlighting the importance of the link between cognitive functions and motor skills. However, most studies have focused on classical spatiotemporal parameters, without addressing an essential feature in pwPD: gait harmonicity. Yet, such harmonicity is fundamental, since it directly reflects some characteristic motor symptoms of PD, such as bradykinesia, freezing of gait, and rhythm variations. An alteration of harmonicity may be a sign of a desynchronization of the underlying motor mechanisms, often accentuated in dual-task conditions. Therefore, our study highlights the importance of exploring not only the quantitative aspects of walking, but also its quality and fluency, particularly in dual-task conditions. This could pave the way for a better understanding of neurocognitive interactions in PD and guide rehabilitation interventions towards a more global and personalized approach. Traditional spatiotemporal gait parameters are informative when considered individually but do not fully capture the global organization of gait. For example, stride time or swing duration provide insight into isolated temporal events, yet they fail to describe how these events integrate into a coordinated locomotor pattern. The *φ*-bonacci gait number addresses this limitation by combining indices of self-similarity, symmetry, and consistency into a unified construct. This integrative approach allows for the detection of subtle disturbances in gait harmonicity that may not be evident when analyzing single parameters independently. Notably, because it reflects the global coordination between motor and cognitive domains, the *φ*-bonacci number may be particularly sensitive to alterations emerging in dual-task conditions. Thus, this metric has the potential to serve as a comprehensive and physiologically meaningful marker of gait harmonicity, extending the interpretative value of traditional spatiotemporal measures. In general, the results that emerged support the hypothesis that the harmonicity of the gait is significantly influenced by the cognitive component, especially in terms of executive and attentive abilities; in fact, they play a fundamental role in maintaining the regularity and harmonicity of the step-in conditions of cognitive load (e.g., the dual task foreseen in the TUG-DT). Our results demonstrate that walking is not a purely automatic process, especially in pwPD, but is strongly modulated by cognitive processes (Yogev-Seligmann et al., 2008). The interaction between motor and cognitive function was further strengthened by the results of the mediation analysis, which showed that the MoCA test, which measures global cognitive function, was a significant negative predictor of the time taken for the TUG-DT, which in turn significantly mediated the effect of cognition on gait harmonicity expressed by the *φ*-bonacci gait number. This result highlights how higher cognitive levels can translate into better motor management under attentional load, indirectly leading to a more harmonious gait. These findings are consistent with previous studies demonstrating that the integration of attention, memory and motor planning is crucial for maintaining postural stability and gait cyclicity in PD (Yogev-Seligmann et al., 2008; Rochester et al., 2014; Maidan et al., 2016; Kehagia et al., 2013). Furthermore, the result of the negative correlation between TUG-DT and MoCA (*r* = −0.536) confirms the sensitivity of TUG-DT in capturing functional cognitive impairment, strengthening its potential as an integrated clinical index in the multidimensional assessment of PD. In parallel, the absence of significant correlations with UPDRS II and III scales suggests that gait harmonicity may not depend solely on motor deficits, but also on alterations of the fronto-striatal circuits implicated in cognitive processes (Maidan et al., 2016). These findings support the view that gait harmonicity is not merely a motor output, but the result of a recursive, self-organizing process governed by principles of temporal symmetrization and self-similarity. A conceptual model (Verrelli et al., 2025) describes movement automatization as unfolding along two interconnected trajectories: the generation of Fibonacci-like temporal patterns via symmetrical coordination, and the progressive refinement of motion through self-similarity mechanisms. This leads to a reduction in Shannon entropy and an increase in movement fluidity and energy efficiency. A conceptual disjunction has been proposed between coordinative mechanization and temporal harmonization, representing two hierarchical levels of automatization. Despite methodological limitations, theoretical insights supported by gait data suggest that the temporal structuring of complex repetitive movements is influenced by cognitive functions such as attention, perception, memory, and decision-making. Although further research is needed to clarify how Shannon entropy minimization translates into neuromuscular optimization, it likely promotes smoother and less costly motor execution. The current findings on the TUG-DT are consistent with previous literature, which highlights how dual-task paradigms can lead to better or worse performance depending on the allocation of attentional resources (Plummer et al., 2013; Leone et al., 2017). In this context, the dual-task cost (DTC) has been proposed as a more appropriate and standardized indicator of motor-cognitive interference. The strong correlation observed between DTC and the *φ*-bonacci value for gait reinforces the main hypothesis of this study, namely that *φ*-bonacci reflects not only the motor component of gait but also its sensitivity to cognitive demands. This supports the idea that the *φ*-bonacci value for gait may represent a comprehensive index of gait harmonicity, particularly under conditions requiring greater cognitive control. Finally, the parameter A1, a self-similarity index of gait, is the main contributor to the value of *φ*-bonacci gait number and this parameter, significantly correlated to the TUG-DT (*r* = 0.605), could represent a biomechanical marker of neurocognitive efficiency. In contrast, the parameters *A*2 and *A*3 although altered in pwPD compared to controls (*p* < 0.05), did not show significant associations with motor performance, suggesting that gait disharmony is primarily driven by the loss of self-similarity captured by *A*1.

### 4.5 Clinical implications

Overall, these results offer new insights into the understanding of gait in PD, highlighting how gait disharmony may represent a tangible manifestation of the impaired interaction between motor control and cognitive function. This has important clinical implications, suggesting that multimodal rehabilitation strategies, oriented not only to motor recovery but also to cognitive enhancement, may improve the effectiveness of interventions on gait quality and fall risk prevention. However, in this context, the absence of specific cognitive outcomes, such as correct responses or errors, is acknowledged, as these would have provided complementary information on performance in dual-task situations and, furthermore, the observed variability may be partly explained by postural strategy, whereby individuals with PD prioritize motor stability over cognitive accuracy in dual-task conditions. Our findings have clinical implications, suggesting that rehabilitation approaches in Parkinson’s disease would benefit from explicitly combining motor and cognitive dimensions. Interventions that integrate gait training with simultaneous cognitive tasks (e.g., dual-task walking, attention or executive function exercises embedded within locomotor training) may improve both gait harmonicity and cognitive efficiency. Recent studies confirm the value of these multimodal strategies, where specific motor exercises are complemented by cognitive stimulation, helping to reduce fall risk and increase functional independence. The *φ*-bonacci gait number could therefore represent an innovative biomarker to guide the personalization and monitoring of motor-cognitive rehabilitation protocols.

## 5 Limitations

This study has some limitations. First, the relatively small sample size reduces statistical power and increases the potential influence of outliers, which should be considered when interpreting the regression results. To address this, we carefully verified all regression assumptions, applied heteroskedasticity-robust standard errors, and performed sensitivity analyses excluding influential cases. Nevertheless, the findings should be regarded as exploratory and hypothesis-generating, requiring validation in larger independent cohorts. Second, although we included an age- and sex-matched healthy control group assessed under identical conditions, further studies on larger samples are warranted to confirm the specificity and generalizability of the *φ*-bonacci gait number in Parkinson’s disease. Given the cross-sectional and observational design, causal inferences cannot be drawn, and the observed association may reflect bidirectional influences, as previously reported in the literature.

Concerning the MOVIT system used for gait analysis, its validation was conducted on a comparatively sample of healthy young adults (*n* = 8, 30 ± 4 years). Although this device has shown good reproducibility in other contexts, further studies including larger and more diverse cohorts of patients with Parkinson’s disease will be necessary to confirm the robustness and generalizability of our findings.

Finally, another limitation is the lack of some measures of cognitive outcomes (e.g., correct responses, errors), which limits the interpretation of the dual-task performance. Furthermore, the postural strategy commonly observed in PD may have influenced participants’ prioritization during dual-task. Future studies should therefore combine motor and cognitive outcomes to provide a more comprehensive assessment of motor-cognitive interference.

## 6 Conclusion

The results of this study confirm the initial hypothesis that gait harmonicity rather than symmetry and consistency in pwPD is statistically significantly influenced by the cognitive component, particularly executive and attentional process. The analysis of the *φ*-bonacci gait number allowed us to quantify innovatively and objectively the consistency, self-similarity, and symmetry during walking, highlighting how gait harmonicity is altered in conditions of cognitive load, such as, in dual-task during gait. The *φ*-bonacci gait number may serve as a novel biomarker to capture motor–cognitive interactions in Parkinson’s disease, offering potential to personalize and monitor integrated rehabilitation strategies aimed at enhancing both gait harmonicity and functional independence. The TUG-DT has proven to be the most sensitive clinical test in detecting these alterations, suggesting that the difficulty in simultaneously managing motor and cognitive tasks reflects a compromise in the integration between the two functional domains. Only the parameter *A*1, reflecting gait self-similarity, emerges as a biomarker of neurocognitive efficiency in pwPD, as its link to Shannon entropy highlights its role in sustaining harmonic locomotor patterns. These findings underline the importance of adopting multimodal rehabilitation strategies that integrate the enhancement of motor skills with interventions aimed at improving cognitive functions, to promote a more harmonious and safe walking, and improve the quality of life of pwPD.

## Data Availability

The raw data supporting the conclusions of this article will be made available by the authors, without undue reservation, upon reasonable request to the corresponding author.
